# Characterization and Clinical Outcome of Philadelphia Chromosome–Positive AML in Thrombocytopenia-Absent Radius Syndrome

**DOI:** 10.1200/PO-24-00411

**Published:** 2024-10-11

**Authors:** Alejandro Villar-Prados, Arman Odabas, Joshua R. Menke, Kerry Kingham, Gabriel N. Mannis

**Affiliations:** ^1^ Division of Hematology, Department of Medicine, Stanford University School of Medicine, Stanford, CA; ^2^ Department of Medicine, Internal Medicine Residency Program, Stanford University School of Medicine, Stanford, CA; ^3^ Department of Pathology, Stanford University, Stanford, CA; ^4^ Department of Genetics, Stanford University, Stanford, CA

## Background

Many insights into genetic mutations that affect hematopoiesis and play a potential role in the pathogenesis of acute leukemia have come from patients with bone marrow failure syndrome (BMFS), as these patients are at increased risk of developing hematological malignancies.^1^ One BMFS that is rarely associated with leukemic transformation is the autosomal recessive thrombocytopenia-absent radius (TAR) syndrome.^2-4^ TAR is characterized by the aplasia or hypoplasia of radii at birth, presence of both thumbs, and hypomegakaryocytic thrombocytopenia that persists through life.^3^ TAR is caused by inherited biallelic variants affecting the *RBM8A* gene.^4,5^ The most common findings are a heterozygous null variant (500-kb deletion or 200-kb deletion) encompassing the *RBM8A* gene at chromosome band 1q21.1 in *trans* with a heterozygous *RBM8A* hypomorphic allele.^4,5^ The protein product of *RBM8A*, Y14, plays a role in exon-junction splicing complex and mRNA splicing.^4,5^ How these heterozygous gene variants contribute to the pathogenesis of acute leukemia is unknown, as only six cases of acute leukemia in patients with TAR are reported in the literature.^2,6-10^

Here, to our knowledge, we present the first known case of a patient with TAR syndrome who developed Philadelphia-chromosome positive (Ph+) AML, which accounts for only about 1% of newly diagnosed AML cases.^11,12^ Written consent was obtained from the patient before preparing and publishing this report.

## Case Presentation

**FIG 1.**
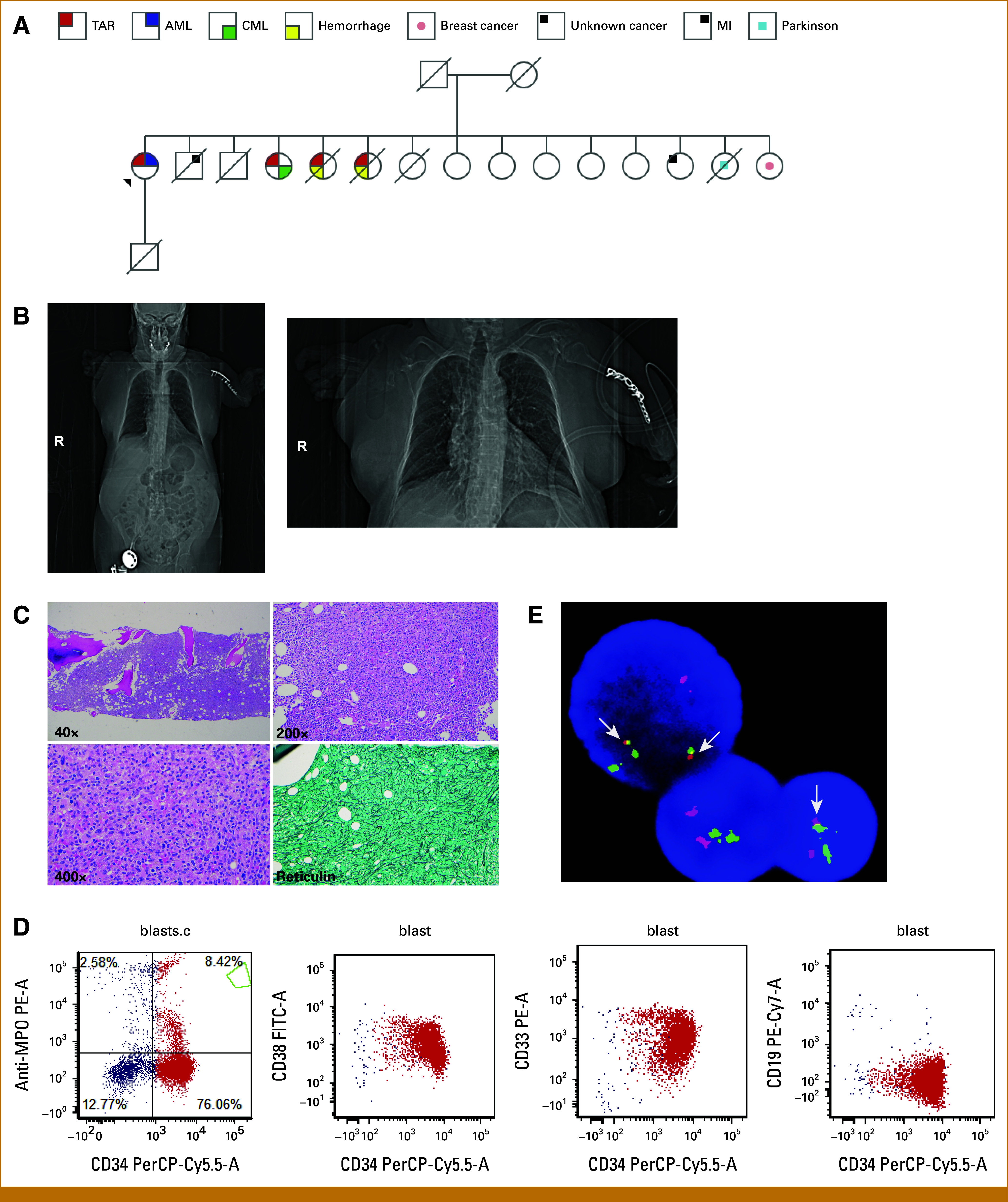
Patient familial history and disease characterization. (A) Family pedigree. (B) Computed tomography scout films of the whole body and chest. (C) Representative hematoxylin and eosin–stained sections from diagnostic bone marrow biopsy core show numerous clusters of blasts, 10:1 myeloid to erythroid ratio with left shifted myeloid maturation, and numerous small megakaryocytes. Reticulin stain, focally severe myelofibrosis, grade 2 to 3 of 3. (D) Representative flow cytometry analysis from diagnostic bone marrow aspirate. (E) FISH for translocation 9;22 (*BCR:ABL*), denoted by white arrows. CML, chronic myelogenous leukemia; FISH, fluorescent in situ hybridization; TAR, thrombocytopenia-absent radius.

The patient is a 71 year-old female with a personal and an extensive family history of TAR (Figs 1A and 1B). Her sister—who also has TAR—was previously diagnosed with chronic myelogenous leukemia (CML). This family was initially described in 1969 by Hall et al,^3^ with no documented hematologic malignancies at that time. She initially presented to a community hospital with symptoms of shortness of breath, cough, daily headache, and weakness. She was found to be pancytopenic and was transferred to our institution because of concern for acute leukemia. Prior available peripheral blood counts from December 2015 to September 2022 did not reveal leukocytosis, neutropenia, basophilia, or anemia with platelet counts ranging from 57 to 126 K/μL.

On admission, pancytopenia was confirmed and was additionally found to have an absolute peripheral blast count of 1.08 K/μL (47.5%). Hematologic laboratory results are summarized in Table 1. Diagnostic bone marrow biopsy and flow cytometry confirmed the diagnosis of AML (FAB classification M2), with moderate to severe fibrosis, grade 2 to 3 (Figs 1C and 1D). Cytogenetic studies demonstrated that 80% of nuclei were positive for a translocation between chromosomes 9 and 22 by fluorescent in situ hybridization (FISH; Fig 1E). This was further confirmed by a somatic targeted next-generation sequencing (NGS) panel developed at Stanford (Heme-STAMP) that covers 203 genes recurrently mutated in myeloid and lymphoid malignancies (Table 1). Reverse transcriptase polymerase chain reaction (RT-PCR) detected the *BCR:ABL1* break point e14a2, consistent with the p210^BCR-ABL^ protein transcript, at >55.0% International Scale (IS). These findings, along with no known history of CML, absence of splenomegaly, and lack of basophilia, supported the diagnosis of AML with *BCR:ABL* fusion by WHO5 classification (Table 1 and Figs 1D and 1E). Array comparative genomic hybridization for chromosome 1q21.1 confirmed a 105 kb deletion. Germline sequencing from cultured skin fibroblasts confirmed both the known 1q21.1 deletion and identified the c.-21 G>A *RBM8A*-mutated hypomorphic allele. No other germline findings were identified among 131 genes associated with hematologic malignancies by NGS (GeneDx Exome Slice). Copy number variant analysis could not be performed on most genes tested.

**TABLE 1.** Summary of Patient Hematologic Laboratory, Molecular, and Genetic Testing

Hematologic Laboratory Tests	Admission, Preinduction Chemotherapy	Day 27 Postinduction (July 24, 23)	Day 80 Postinduction (September 8, 23)	Day 307 Postinduction (May 2, 24)
Complete blood count (CBC) with differential				
Total white blood cell count (K/µL)	2.3	0.5	14.6	9
Hemoglobin (g/dL)	8.8	8.8	9.3	11.7
Hematocrit (%)	26.4	25.9	29.3	36.4
Platelet count (K/µL)	58	57	3	30
MCV (fL)	86.8	84.9	88.8	82.9
RDW (%)	20.4	17.9	17.1	16.5
Neutrophils (%)	11	Below detection threshold	86	79.5
Band neutrophils (%)	3	Below detection threshold	0	0
Metamyelocytes (%)	1	Below detection threshold	0	0
Lymphocytes (%)	33.5	Below detection threshold	3.7	12.5
Monocytes (%)	2	Below detection threshold	6.5	3.6
Basophils (%)	2	Below detection threshold	0.9	1.8
Eosinophils (%)	0	Below detection threshold	1.9	2.2
Blast (%)	47.5	Below detection threshold	0	0
Coagulation laboratory results				
D-Dimer (µg/mL FEU)	0.92	3.02	0.82	—
Fibrinogen (mg/dL)	309	508	386	—
Prothrombin time (seconds)	14.8	19.4	13.8	—
Partial thromboplastin time (seconds)	29.4	34.6	35	—
INR	1.1	1.6	1	—
Bone marrow biopsy	Diagnostic bone marrow biopsy on day 4 of admission	Repeat bone marrow biopsy on day 30 of admission	—	—
Cellularity (%)	90	30	—	—
Myeloid-to-erythroid ratio	10:1	3:1	—	—
Bone marrow blast (%)	50	<5	—	—
Phenotypic flow cytometry markers	Positive for CD34, CD38, CD13, CD33, CD117 (dim to moderate bright), HLA-DR, CD45 (dim) and MPO (minor subset). Negative for CD3, CD19, among others)	0.40% positive population CD34, CD38, CD13, CD33, CD117, HLA-DR, and CD45 (dim)	—	—
HemeSTAMP (next generation sequencing)	Positive BCR:ABL1 fusion. *STAT5B* (Tyr392Asn, VAF 50%); *PPM1D* (p.Ile496Val, VAF 50%); *CREBBP* (p.Val238Leu, VAF 49%); *IKZF2* (p.Leu328Phe, VAF 46%)^a^	*STAT5B* (Tyr392Asn, VAF 48%); *PPM1D* (p.Ile496Val, VAF 50%); *CREBBP* (p.Val238Leu, 53%); *IKZF2* (p.Leu328Phe, 48%)^a^	—	—
Karyotype	46,XX,t(9:22) (q34;q11.2)[1]/46XX[1]	46XX,t(9:22) (q34,q11.2)[1]/46XX[1]	—	—
Cytogenetics	Positive for *BCR:ABL1* t(9;22), 80.5% of cells. Negative for del(5q), del(97q), trisomy 8, del(20q)	Positive *BCR:ABL1*, 11% of cells	—	—
Additional testing				
BCR-ABL p210 RT PCR transcript levels (International Scale)	>55%	7%	0%	0%
Additional molecular testing	Negative for *NPM1*, *FLT3-ITD*, *FLT3-D835*, *IDH1* and *IDH2* mutations	Not performed	Not performed	Not performed

Abbreviations: MCV, mean corpuscular volume; RDW, red blood cell distribution width; RT PCR, reverse transcriptase polymerase chain reaction; VAF, variant allelic frequency.

^a^ Mutations detected by somatic next-generation sequencing from bone marrow biopsies in *STAT5B*, *PPM1D*, *CREBBP*, and *IKZF2* are of unknown clinical significance. See discussion.

Given the patient's age and poor functional status, the induction regimen consisted of decitabine (20 mg/m^2^ once daily, days 1-5), venetoclax 100 mg once daily (dose reduced from 400 mg while receiving posaconazole for neutropenic prophylaxis, days 1-14), and dasatinib 100 mg once daily.^13^ Dasatinib was eventually dose adjusted to 50 mg once daily because of severe diarrhea. Day+ 27 postinduction bone marrow biopsy revealed <5% blasts consistent with complete morphologic response with incomplete platelet recovery (CRi). FISH showed decreased positivity for t(9;22) at 11% and decreased p210^BCR-ABL^ transcript at 7% IS (Table 1). She was discharged on day+ 38. Two months after discharge, p210^BCR-ABL^ RT-PCR was undetectable, and she remained in CRi. The patient received only one cycle of venetoclax with decitabine because of poor tolerability. Given her CRi and after discussion with the patient, we opted to forgo additional chemotherapy cycles and just continue dasatinib maintenance alone, start eltrombopag for platelet support, and continue frequent laboratory monitoring**.** Because of diarrhea, she was transitioned from dasatinib to imatinib monotherapy (day+ 237 postinduction). She was not an allogeneic bone marrow transplant candidate.

## Discussion

Acute leukemia is rarely observed in TAR, with no previously reported cases of Ph+ AML.^2,6-10^ From the available cases, AML is the most prevalent leukemia (Table 2). Of the reported AML cases, patients were significantly younger when compared with our patient^2,6,9,10^ (Table 2). From the adult patients with AML, one developed secondary AML from myelodysplastic syndrome with a *CALR* mutation^2^ while the other had de novo disease with a t(8;21) translocation.^9^ Of all the reported patients, only Beauvais et al^7^ confirmed both micro-deletion 1q21.1 and germline mutation of RBM8A in a patient with T-cell acute lymphoblastic leukemia. Like Go and Johnston,^9^ our patient had a favorable response to treatment, achieving CRi and transfusion independence. Our patient's persistent thrombocytopenia after treatment is consistent with previous reports and was expected in the setting of TAR with ongoing tyrosine kinase treatment (TKI).^7,9^

**TABLE 2.** Summary of Reported Patients With TAR With Acute Leukemia

Publication	Presented Case	Camitta and Rock^9^	Rao et al^7^	Fadoo and Naqvi^11^	Go and Johnston^10^	Jameson-Lee et al^2^	Beauvais et al^8^
Age	71 year	5 year	2 months	13 months	42 year	47 year	20 year
Sex	Female	Female	—	Male	Female	Male	Female
Physical examination	Bilateral radial and ulnar aplasia with right humeral hypoplasia, normal thumbs	Short forearms, bilateral radial aplasia, normal thumbs	Absence of upper radii, normal thumbs	Bilateral absence of upper arms and forearms, normal thumbs, bilateral tibial torsion	Short forearms, bilateral radial aplasia, normal thumbs	Bilateral upper extremity phocomelia	Bilateral absent radii, bilateral hip dislocation, normal thumbs
Family history	4 siblings with TAR. 1 female sibling with TAR and CML	1 sister with TAR	—	No TAR history	—	—	—
Disease	AML	B-ALL	AML	AML	AML	AML (progressed from MDS)	T-ALL
Cytogenetics	46,XX,t(9:22) (q34;q11.2)[1]/46XX[1]	Complex, 46/57 XX (+10,+14,+21,+6 and +X)	—	—	t(8;21) (q22;q22)	Add (3p) and add (12q)	46,XX [7]/97,XXXX,+1,+mar,var [5]. 347kb micro deletion of chromosome 1q21.1
Somatic genetic and molecular testing	Positive BCR:ABL1 fusion. 105 kilobase (kb) deletion on chromosome 1.21.1 on comparative genomic hybridization from peripheral blood	—	—	—	—	*CALR*	Biallelic CDKN2A (9p21.1) deletion; biallelic ETV6 (12p13.2) deletion; TLX1 overexpression; NOTCH1 mutation
Germline *RBM8A* mutation testing (fibroblast)	1q21.1 deletion (heterozygous). RBM8A c.-21 G>A (heterozygous)	—	—	—	—	—	Heterozygous deletion RBM8A
Treatment course	Induction with decitabine and venetoclax and dasatinib. Received 1 cycle. Kept on TKI maintenance, first with dasatanib and transitioned to imatinib	4 weeks induction prednisone, vincristine, asparaginase with 24 weeks intensification of methotrexate and 6-mercaptopurine	—	None, pursued palliative treatment after AML diagnosis	Cytarabine and idarubicin (7 + 3) plus 3 cycles of high dose cytarabine	Decitabine priming with subsequent cytarabine and idarubicin (7 + 3). Required second induction 7 + 3	FRALLE 2000-T regimen at induction with 14 month maintenance with 6-mercaptopurine, vincristine, methotrexate and prednisone
Clinical outcome	CRi with incomplete platelet recovery. Persistent thrombocytopenia, with no bleeding complications or requiring platelet transfusions	CR2, planned for unrelated allogeneic bone marrow transplant	—	Death shortly after diagnosis	CRi with incomplete platelet recovery	Morphologic CR with persistent molecular disease. Underwent matched related donor allogeneic bone marrow transplant	CR with complete molecular response. Delayed platelet recovery after each chemotherapy cycle

Abbreviations: CML, chronic myelogenous leukemia; CR, complete remission; TAR, thrombocytopenia-absent radius; TKI, tyrosine kinase treatment.

Germline and somatic mutations associated with marrow hypocellularity are known to increase the risk of myeloid malignancies.^14^ In TAR, biallelic pathogenic variants in *RBM8A* are suggested to impair megakaryocyte differentiation.^15^ Increasing evidence also supports how genetic defects in spliceosome and RNA binding proteins drive the progression of myeloid malignancies.^16^ Loss of function of *RBM8A* may provide the correct conditions within the hematopoietic stem cell populations to drive myeloid malignancies. In addition to mRNA splicing, Y14 has been suggested to aid in the repair of DNA double-strand breaks in vitro.^17^ In this context, loss of Y14 could increase the propensity for DNA double-strand breaks and promote chromosomal translocations, including *BCR::ABL1*. This hypothesis may be supported by the patient's family history of TAR and CML, although further molecular studies are needed.

Somatic NGS from the bone marrow identified four incidental point mutations of unknown significance in *STAT5B*, *PPMD1*, *IKZF2*, and *CREBBP*, at a 50% variant allelic frequency (VAF) which have not been reported previously in TAR or Ph+AML.^4,18^ To further investigate these mutations, we queried the NCBI ClinVar database.^19^ *PPMD1* (p.Ile496Val) and *CREBBP* (p.Val238Leu) were cataloged in ClinVar and predicted to be likely benign. *IKZF2* (p.Leu328Phe) and *STAT5B* (p.Tyr392Asn) were not found in ClinVar. Regardless, these mutations may have been of significance. *STAT5B* is a known downstream signaling target of *BCR::ABL1*.^20^ The identified *STAT5B* mutation corresponds to the DNA-binding domain, which has not been described in Ph+AML. For *IKZF2,* the identified mutation did not map to a known protein domain, thus the biological impact of this mutation is uncertain. The 50% VAF of these four gene mutations suggests that they may be germline, but these were not assessed in the germline GeneDx NGS since these genes are not included in that panel. Although it may be helpful to gain further insights into the etiology of leukemic transformation in Ph+ AML and/or TAR, at this time, we do not have somatic NGS testing from the patient's sibling to determine whether she shared these mutations. Despite the presence of these incidental mutations, the patient has remained in remission with single-agent TKI maintenance, suggesting *BCR::ABL1* is the main disease driver.

To our knowledge, this is the first documented patient with Ph+AML TAR diagnosed at such an advanced age and treated with lower intensity induction with venetoclax, decitabine, and dasatinib with TKI maintenance. Our treatment approach, although not novel, proved effective in this patient. The favorable outcome is consistent with a large retrospective report of patients with AML harboring mutations in spliceosome genes treated with lower-intensity regimens containing venetoclax.^21^ This case also supports synergy of *BCL2* inhibition with venetoclax and *BCR::ABL1* inhibition in myeloid malignancies.^13^ Given the low number of patients with TAR diagnosed with AML, it is difficult to determine if this approach can be extrapolated to other patients with TAR, warranting future clinical studies.

A limitation of our report is that despite the patient's initial presentation, we cannot completely rule out CML with myeloid blast crisis. We do not have life-long blood counts for our patient, but with the available data in our medical record, she did not have a period of sustained leukocytosis or basophilia. In a study by Soupir et al,^12^ the absence of splenomegaly and basophilia favors Ph+AML, whereas high cellularity and M:E ratio is more suggestive of prior CML .

This report highlights Ph+AML as a rare and potential complication associated with TAR syndrome and a promising treatment strategy for older, unfit patients with TAR Ph+AML. Future molecular and genetic studies are needed to determine how genes in the 1q21.1 locus, including *RBM8A,* may be implicated in hematopoiesis and leukemic transformation.
